# Platelet–neutrophil interactions as drivers of inflammatory and thrombotic disease

**DOI:** 10.1007/s00441-017-2727-4

**Published:** 2017-11-25

**Authors:** Ton Lisman

**Affiliations:** Surgical Research Laboratory and Section of Hepatobiliary Surgery and Liver Transplantation, Department of Surgery, University of Groningen, University Medical Center Groningen, BA33, Hanzeplein 1, 9713 GZ Groningen, The Netherlands

**Keywords:** Platelet, Neutrophil, Inflammation, Thrombosis, Ischemia

## Abstract

Neutrophils are well known for their role in infection and inflammatory disease and are first responders at sites of infection or injury. Platelets have an established role in hemostasis and thrombosis and are first responders at sites of vascular damage. However, neutrophils are increasingly recognized for their role in thrombosis, while the immunemodulatory properties of platelets are being increasingly studied. Platelets and neutrophils interact during infection, inflammation and thrombosis and modulate each other’s functions. This review will discuss the consequences of platelet–neutrophil interactions in infection, thrombosis, atherosclerosis and tissue injury and repair.

## Introduction

Platelets are anuclear cell fragments that circulate in healthy humans at about 150,000–400,000 per microliter blood. Platelets are well known for their role in thrombosis and hemostasis. Inherited or acquired defects in platelet count and/or function can be associated with bleeding complications. Conversely, platelets are key players in thrombotic disease and drugs inhibiting platelet function are vital in treatment and prevention of arterial thrombotic diseases such as myocardial infarction and stroke. Recent studies show that platelets have many functions beyond physiological or pathological thrombus formation. They play a role in inflammation, are effectors of injury in a variety of pulmonary disorders and syndromes, facilitate tissue repair and act in the growth and development of metastases of various cancers (Xu et al. 2016). Although platelets can exert some of their functions individually, it is increasingly recognized that interactions between platelets and other circulating cell types are crucial for many of their functions. In this review, I will address the interaction of platelets with neutrophils and the role of this interaction in inflammation and infection, thrombosis, atherosclerosis and tissue injury and repair.

## Platelet-neutrophil complexes

It has been long known that circulating platelet–neutrophil complexes are present in a wide range of inflammatory conditions including bacterial infections and sepsis (Gawaz et al. 1995), inflammatory bowel disease (Pamuk et al. 2006), sickle cell disease (Polanowska-Grabowska et al. 2010), atherosclerosis and coronary syndromes (Ott et al. 1996; Maugeri et al. 2014) and pulmonary inflammatory syndromes (Gresele et al. 1993; Caudrillier et al. 2012). Platelets interact with neutrophils by multiple interactions including platelet P-selectin binding to neutrophil P-selectin glycoprotein ligand-1 (PSGL-1) (Hamburger and McEver 1990; Moore et al. 1995) and platelet glycoprotein Ibα binding to neutrophil MAC-1 (Simon et al. 2000). P-selectin exposure on the platelet surface requires platelet activation. Indeed, platelet–neutrophil complexes have been used as markers for platelet activation and, surprisingly, these complexes appear better markers of in vivo platelet activation compared to direct assessment of platelet P-selectin expression (Michelson et al. 2001). The generation of platelet–neutrophil complexes in vivo is facilitated by margination of platelets and neutrophils to the periphery of blood vessels as a consequence of displacement of erythrocytes to the central part of the vessels (Goldsmith and Spain 1984; Goldsmith et al. 1999). This margination process greatly enhances the possibility of collisions between neutrophils and platelets. Stable platelet–neutrophil complexes require platelet activation that may occur in solution but could also occur as a result of platelet adhesion to activated endothelial cells or a vascular injury. Platelets are capable of adhering to activated or injured endothelium from flowing blood and the efficacy of platelet adhesion actually increases with increasing shear rate (Remuzzi et al. 1985; Houdijk et al. 1986). The unique biophysical properties of the interaction of platelet glycoprotein Ibα with collagen- or endothelial cell-bound von Willebrand factor (VWF) underlie shear stress-mediated enhancement of platelet deposition (Ciciliano et al. 2015). In contrast, neutrophils, although able to adhere to activated endothelial cells, become less efficient in adhering with increasing shear (Kuijper et al. 1996). Platelets therefore facilitate neutrophil adhesion to activated or injured endothelium at higher shear and thereby promote leucocyte transmigration across the endothelium. A recent study showed that neutrophil adhesion to activated platelets proceeds via long membrane protrusions that extend from adhered platelets and are formed under conditions of flow both in vitro and in vivo (Tersteeg et al. 2014). These elongated membrane structures (referred to as flow-induced protrusions or FLIPRs) were shown to bind neutrophils in a P-selectin/PSGL-1-dependent manner. This interaction led to neutrophil activation and transfer of microparticles from the FLIPR to the neutrophil. Interestingly, the authors suggested that neutrophil–platelet microparticle complexes may also occur in vivo, and that part of the previously reported neutrophil–platelet complexes are in fact neutrophil–platelet microparticle complexes. In the following sections, I will address functional consequences of platelet–neutrophil interactions.

## Platelet–neutrophil interactions in inflammation and infection

Neutrophils are key first responders to sites of injury and infection and are increasingly recognized as actors in chronic inflammatory states (Deniset and Kubes 2016; Soehnlein et al. 2017). Neutrophils combat invading pathogens by a combination of phagocytosis, generation of reactive oxygen species and release of neutrophil extracellular traps (NETs). The platelet–neutrophil interplay is key in all these inflammatory responses, as it helps localizing platelets to inflammatory sites, potentiates production of oxygen radicals and is key in neutrophil activation to release NETs (Page and Pitchford 2013).

It has been shown that neutrophils continuously patrol the vasculature for activated platelets to initiate inflammatory responses (Sreeramkumar et al. 2014). Activated platelets are crucial in neutrophil-mediated inflammatory responses, as depletion of either cell type decreases mortality in models of pathological inflammation including acute lung injury (Looney et al. 2009) and sepsis (Sreeramkumar et al. 2014). Interactions between P-selectin and PSGL-1 and glycoprotein Ibα and MAC-1 have been shown to drive platelet–neutrophil interactions under inflammatory conditions (Sreeramkumar et al. 2014; Wang et al. 2017). Interestingly, while depletion of platelets decreases neutrophil recruitment under inflammatory conditions, the reverse is also true (Clark et al. 2007; Sreeramkumar et al. 2014). Thus, the observation that platelet influx is also decreased when neutrophils are depleted suggests that the model that neutrophils scan inflammatory sites for activated platelets might be too simplified.

Besides a role in neutrophil localization, activated platelets initiate or amplify various neutrophil responses including phagocytosis and production of oxygen radicals and production of NETs. Such responses are initiated by direct contact but also by release of soluble mediators such as CCL5 and platelet factor 4 (von Hundelshausen et al. 2005; Pervushina et al. 2004). Conversely, neutrophils also release soluble mediators such as cathepsin G and elastase that enhance platelet responses by activation of protease-activated receptors on platelets (Selak 1992; Sambrano et al. 2000; Mihara et al. 2013). Interestingly, these neutrophil-derived enzymes are also negative regulators of platelet adhesion as they may proteolyse VWF (Bonnefoy and Legrand 2000).

Platelet interactions have been shown to enhance the phagocytic capacity of neutrophils towards various bacteria in vitro (Assinger et al. 2011; Peters et al. 1999; Hurley et al. 2015). In addition, thrombocytopenia increases the bacterial load in animal models of bacterial infection (de Stoppelaar et al. 2014; van den Boogaard et al. 2015). In addition, platelets may enhance the release of reactive oxygen species and myeloperoxidase further contributing to pathogen killing (Zalavary et al. 1996; Gros et al. 2015b). However, under certain experimental conditions, platelets down-regulate the oxidative burst and the action of platelets in the inflammatory response and therefore appears context-dependent (Lecut et al. 2012). Platelets are also key in the formation of neutrophil extracellular traps (NETs) (von Bruhl et al. 2012; Clark et al. 2007). NETs are extracellular webs generated upon neutrophil activation, consisting of neutrophil DNA with various proteins such as histones attached (Brinkmann et al. 2004). NETs capture and kill pathogens directly and inactivate virulence factors by elastase present within the NET. Platelet-mediated NET formation requires platelet–neutrophil interactions by P-selectin/PSGL-1 interactions or by binding of neutrophil β2 integrins with GPIbα or αIIbβ3 on the platelet (Jenne et al. 2013; Etulain et al. 2015; Caudrillier et al. 2012; Carestia et al. 2016). In experimental sepsis models, activation of platelet TLR4 was shown to be key in the generation of NETs (Clark et al. 2007). Furthermore, platelets stimulate neutrophil transmigration over the endothelium, likely by a combination of assisting neutrophil adhesion to the endothelium and enhancing endothelial cell permeability (Gros et al. 2015a). Finally, platelets stimulate the formation of various leukotrienes by neutrophils in an intriguing mechanism involving transfer of the arachidonic acid metabolite 12-HETE from platelet to neutrophils, which further process this molecule to bioactive leukotrienes (Marcus et al. 1984, 1987, 1988). This transcellular arachidonic acid pathway presumably requires platelet–neutrophil contact given the key role of P-selectin in this process (Maugeri et al. 1994). Leukotrienes such as LTC4, LTD4, LTE4, LTB4 and 12- and 20-diHETE have various downstream effects including enhancement of vascular permeability, modulation of smooth muscle cell contractility and chemo-attraction (Peters-Golden and Henderson 2007). Interestingly, the transcellular arachidonic acid metabolism is bidirectional, as platelets also use arachidonic acid released from neutrophils to increase 12-HETE production (McCulloch et al. 1992).

## Platelet–neutrophil interactions in thrombosis

Animal models of thrombosis have demonstrated a key role of neutrophils in venous and arterial thrombosis. In a model of venous thrombosis induced by flow restriction, leukocytes (predominantly neutrophils) have been shown to crawl along and adhere to the surface of (activated) endothelial cells, eventually forming an almost continuous layer on the vascular endothelium (von Bruhl et al. 2012). These leukocytes have been demonstrated to initiate and propagate venous thrombosis in this model. Similarly, neutrophils have been shown to be the first responding cells in response to arterial injury induced by a laser (Darbousset et al. 2012). However, in other models, platelets were the first responding cells, with leukocyte influx critically dependent on deposited platelets (Palabrica et al. 1992; Gross et al. 2005). Although it has been long recognized that leukocytes contribute to activation of coagulation in thrombosis, the mechanisms involved are still incompletely understood. Importantly, a recent study using a mouse model of spontaneous venous thrombosis found that neutrophil depletion did not affect thrombosis development despite the observation that neutrophils were abundant within the thrombus, indicating that the role of neutrophils in thrombosis may be context-dependent (Heestermans et al. 2016).

At least three distinct mechanisms may be involved in platelet-dependent, neutrophil-mediated induction of thrombosis. First, neutrophils have been demonstrated to transfer tissue factor, the physiological initiator of coagulation, to platelets during thrombus formation in vitro (Giesen et al. 1999). This transfer proceeds via TF-containing microparticles derived from neutrophils and is dependent on TF and microparticle CD15 (Rauch et al. 2000). Also in vivo, TF-bearing microparticles have been shown to be incorporated into thrombi early after vascular injury and to drive thrombus formation (Gross et al. 2005). During thrombus formation, TF requires ‘decryption’, i.e., transformation from a circulating non-coagulant, to a thrombus-associated coagulant form (Chen and Hogg 2013). Decryption requires thiol isomerase activity by, for example, protein disulfide isomerase.

The ‘blood-borne’ TF concept challenges the dogma that tissue factor is normally not located on cells in contact with blood and the source and quantity of blood borne TF is subject to ongoing debate (Butenas et al. 2005; Cimmino et al. 2011). For example, while some have found that platelets contain TF (Muller et al. 2003; Schwertz et al. 2006; Panes et al. 2007; Camera et al. 2003), others failed to reproduce these findings (Bouchard et al. 2010; Osterud and Olsen 2013). Distinct mechanisms for platelet TF expression have been proposed: (1) acquisition from leukocyte-derived microparticles (Giesen et al. 1999), (2) storage in platelet α granules (Muller et al. 2003) and (3) de novo synthesis from mRNA stored within the (anucleate) platelet (Schwertz et al. 2006). However, all these findings have been questioned with reference to an inadequate methodology used in these studies. Similarly, the finding that neutrophils synthesize and may transfer tissue factor (Giesen et al. 1999) has been challenged (Osterud et al. 2000). Nevertheless, it is likely that blood-borne TF contributes to initiation and propagation of thrombosis given protection of mice lacking TF specifically on myeloid cells (von Bruhl et al. 2012). Furthermore, the association between thrombotic diseases and elevated levels of TF-bearing microparticles in blood are consistent with a role of blood-borne TF in thrombosis (Zwicker et al. 2011).

A second mechanism by which the platelet–neutrophil axis contributes to initiation and propagation of thrombosis is through generation of NETs (Fuchs et al. 2010; Brill et al. 2012). NETs have been directly implicated in thrombosis as prevention of NET formation using mice deficient in PAD4 (Martinod et al. 2013), which cannot undergo the histone modification required for chromatin decondensation in NET formation and disintegration of NETs by DNAse (Fuchs et al. 2010) reduced the thrombus load in mouse models. Also, NETs have been demonstrated in human thrombi (Stakos et al. 2015; Savchenko et al. 2014) and NET components including nucleosomes, histones and cell-free DNA are elevated in patients with thrombotic disease (van Montfoort et al. 2013; Jimenez-Alcazar et al. 2017), although it has not been established whether this is a consequence of the event or that in humans elevated NET components indicate a risk for a future thrombotic event. NETs appear to drive thrombus formation by multiple mechanisms including TF-mediated initiation of coagulation, FXII-mediated initiation of coagulation, adhesion of platelets, recruitment of platelet adhesive proteins such as VWF, recruitment of red blood cells and inhibition of clot breakdown (Martinod and Wagner 2014; Gould et al. 2015). Although it has been well established that NETs support thrombin generation in vitro and in vivo, the mechanisms involved are incompletely understood and partly controversial. A recent study found that NET components but not NETs themselves, are procoagulant in vitro (Noubouossie et al. 2017). Indeed, a number of studies have shown activation or amplification of coagulation by cell-free DNA but part of these results have been challenged as procoagulant effects of isolated DNA appear to be related to activators co-purified with the DNA, notably silica particles (Smith et al. 2017). In addition, histones have been shown to drive thrombin generation and inhibit protein C-mediated anticoagulant responses (Ammollo et al. 2011).

A third mechanism linking the platelet–neutrophil axis to thrombosis relates to neutrophil constituents modulating hemostasis. For instance, neutrophil cathepsin G and elastase inactivate natural anticoagulant systems including tissue factor pathway inhibitor, thrombomodulin and antithrombin (von Bruhl et al. 2012; MacGregor et al. 1997; Jordan et al. 1989). In addition, neutrophil oxidants inactivate thrombomodulin and ADAMTS13 (Glaser et al. 1992; Wang et al. 2015), while VWF modification by neutrophil oxidants renders it resistant to ADAMTS13 cleavage (Chen et al. 2010). Finally, neutrophil peptides (α defensins) have also been shown to inhibit VWF cleavage by ADAMTS13 (Pillai et al. 2016). Thus, inactivation of key anticoagulant systems and inhibition of ADAMTS13-mediated regulation of VWF-dependent thrombus formation all contribute to procoagulant effects of neutrophils. However, neutrophil cathepsin G and elastase are able to proteolyse VWF, which may compensate for the defect in ADAMTS13-mediated cleavage (Bonnefoy and Legrand 2000; Raife et al. 2009).

## Platelet–neutrophil interactions in atherosclerosis

Platelets have long been implicated in acute coronary events. Their key role is evidenced by the success of platelet inhibitory drug in treatment and prevention of acute arterial thrombotic events (Jamasbi et al. 2017). Additionally, platelets have been implicated in atherogenesis and platelet adhesion to activated endothelium is a very early event in the process of atherogenesis and drives subsequent proinflammatory responses leading to plaque formation (Gawaz et al. 2005). Attraction of leukocytes, particularly monocytes, to the developing atherosclerotic lesion has also long been recognized as a key event in atherogenesis (Gistera and Hansson 2017). Platelets facilitate monocyte adhesion to and transmigration through the endothelial cell layer and facilitate the formation of foam cells from these macrophages. The role of the neutrophil in atherogenesis has long escaped attention due to the pivotal role of monocytes in the process. However, several lines of evidence suggest neutrophils and platelet–neutrophil interactions to also be key players in atherogenesis and acute coronary events. Firstly, neutrophil counts are predictors of future coronary events (Shah et al. 2017; Horne et al. 2005) and local neutrophil accumulation is associated with the outcome of a coronary event (Distelmaier et al. 2014). Secondly, animal models have shown NET formation in the developing atherosclerotic lesion (Warnatsch et al. 2015), while inhibition of PAD4 (which is required for NET formation) decreased the size of the atherosclerotic lesion indicating that NET formation is a driver of atherogenesis (Knight et al. 2014). Indeed, in humans, NET components have been associated with the severity of atherosclerosis and the future risk of cardiac events (Borissoff et al. 2013). Finally, NETs have been identified in coronary thrombi obtained during thrombectomy or from patients with stent thrombosis (Mangold et al. 2015; Riegger et al. 2016). It is incompletely understood how NETs drive atherogenesis but mechanisms may include activation of plasmacytoid dendritic cells resulting in a type I interferon response (Doring et al. 2012), activation of macrophages that subsequently release inflammatory cytokines (Warnatsch et al. 2015), or enhancement of endothelial dysfunction (Carmona-Rivera et al. 2015).

## Platelet–neutrophil interactions in tissue injury and repair

Sterile tissue injury as for example encountered in injury by toxins (Miyakawa et al. 2015), ischemia/reperfusion injury (Tamura et al. 2012), or sterile traumatic injury (Slaba et al. 2015) results in recruitment of platelets in response to endothelial injury. Neutrophils are also recruited to sites of sterile injury and consequent platelet–neutrophil interactions will contribute to exacerbation of injury or to repair, depending on the context.

For example, acetaminophen-induced liver injury results in an influx of platelets and neutrophils in the liver microcirculation (Miyakawa et al. 2015). Depletion of either platelets or neutrophils decreases hepatocellular injury (Liu et al. 2006; Miyakawa et al. 2015). As depletion of platelets also decreases neutrophil influx, it may be that platelet–neutrophil crosstalk is involved in driving acetaminophen-induced liver injury. Of note, although it has been demonstrated that neutrophil depletion decreases acetaminophen-induced hepatocellular injury, these results have been questioned (Jaeschke and Liu 2007) and it has been suggested that neutrophils do not directly contribute to injury but are only involved in tissue repair following acetaminophen intoxication (Williams et al. 2014). Similarly, platelets have been shown to drive neutrophil-mediated liver damage following acute alpha-naphthylisothiocyanate intoxication (Sullivan et al. 2010).

Platelets and neutrophils have been implicated as drivers of ischemia/reperfusion injury in liver (Yadav et al. 1999), kidney (Jansen et al. 2017), heart (Bonaventura et al. 2016) and lungs (Sayah et al. 2015) and platelet–neutrophil crosstalk has been suggested to be an important contributor of injury. Recent studies have shown NET formation to contribute to ischemia/reperfusion injury as inhibitors of NET formation or NET degradation by DNAse reduced injury in experimental animal models (Nakazawa et al. 2017; Sayah et al. 2015; Ge et al. 2015; Huang et al. 2015). Such studies indicate that NETs are not only involved in infection and thrombosis but also reinforces the notion that NETs drive non-infectious, non-thrombotic disease (Jorch and Kubes 2017). The exact mechanisms by which NETs drive ischemia/reperfusion injury are unknown but part of the mechanism may involve intravascular thrombus formation, as inhibitors of coagulation activation have also been shown to reduce ischemia/reperfusion injury (Okajima et al. 2002; Tillet et al. 2015). Studies in humans have also shown the formation of NETs in transplanted lungs and it has been suggested that NET formation could lead to acute graft failure (Sayah et al. 2015).

Platelets trigger the release of NETs in a model of experimental transfusion-associated acute lung injury and thereby aggravate disease (Caudrillier et al. 2012). Pulmonary injury was substantially decreased by NET degradation by DNAse, platelet inhibition or an anti-histone antibody. Importantly, these interventions improved survival from ~50% to 100% in this model.

Platelet–neutrophil interactions have been shown to facilitate repair in a model of heat-induced liver injury (Slaba et al. 2015). Specifically, it was shown that platelets immediately attached to the endothelium adjacent to the site of injury in a αIIbβ3-dependent manner. These platelets were required for subsequent neutrophil influx and these neutrophils assisted in the wound-healing response. Importantly, very few NETs were generated in this model, showing that platelet–neutrophil interactions that are physiologically relevant do not necessarily lead to formation of NETs.

Platelets are well known to be required for liver regeneration following partial hepatectomy and it has been suggested that growth factors stored within platelet granules drive platelet-mediated liver regeneration (Lesurtel et al. 2006; Starlinger and Assinger 2016; Starlinger et al. 2014; Matsuo et al. 2008). However, since both neutrophils and platelets are required for liver regeneration following partial hepatectomy (Selzner et al. 2003; Lesurtel et al. 2006), it has been suggested that the mechanism by which platelets stimulate liver regeneration may be through leukocyte recruitment, similar to that demonstrated in the model of thermal liver injury (Lisman and Porte 2016).

Finally, NETs have also been demonstrated to contribute to organ injury in sepsis (Czaikoski et al. 2016), which may in part be due to deleterious effects of NET formation and subsequent intravascular coagulation on the microcirculation (McDonald et al. 2017). Treatment with DNAse also reduced organ damage in this setting (Czaikoski et al. 2016).

## The platelet–neutrophil interaction: a therapeutic target?

Experimental and clinical evidence for a role of platelet–neutrophil crosstalk in a variety of clinical conditions including inflammation, thrombosis, atherosclerosis and tissue injury is rapidly emerging. Agents blocking PSGL-1, P-selectin, or MAC-1 that have pleiotropic effects that include inhibition of platelet–neutrophil interactions have been trialed in humans (Mertens et al. 2006; Stahli et al. 2016; Jones 2000) but are no longer in clinical development as clinical studies did not show a benefit of these agents. Given the key role of NETs in many clinical situations in which the platelet–leukocyte interaction is involved, it may be that inhibitors of NET formation or agents aimed at dissolving NETs could reignite the interest in agents aimed at blocking platelet–neutrophil interplay. Indications for such agents could include venous and acute arterial thrombosis, disseminated intravascular coagulation and various types of NET-mediated organ injury such as ischemia/reperfusion injury and transfusion-associated acute lung injury. In considering targeting NETs therapeutically, it should be remembered that NETs are likely required for fighting severe infection. It has been suggested that agents targeting NETs would be safe in the absence of overt infection but that concomitant administration of antibiotics would be indicated in cases of an infection (Martinod and Wagner 2014). Other diseases in which NETs have been implicated but which have not been discussed in this review include systemic lupus erythematosus, rheumatoid arthritis, diabetes, vasculitis and cancer (Jorch and Kubes 2017). Whether pharmacological targeting of NETs will be beneficial in these chronic diseases is an open question but clearly the risk of infection may hamper chronic use of such agents.

Inhibitors of PAD4 may have merit given the key role of PAD4 in NET formation and the intact NET-independent anti-inflammatory properties of PAD4-deficient neutrophils (Li et al. 2010; Martinod and Wagner 2014). Alternatively, DNAseI, which has been shown to efficiently clear thrombi and decreased NET-mediated injury in experimental animal models may have merit, particularly since DNAseI is an FDA-approved drug (Pulmozyme, inhaled using a nebulizer for cystic fibrosis) and has also been trialed as an intravenous drug (Davis et al. 1999).

A clear advantage of targeting NETs in thrombotic syndromes is the lack of effect of the intervention on physiological hemostasis. As bleeding is an important side effect of current therapies for venous and arterial thrombosis and disseminated intravascular coagulation, the development of agents with a better risk/benefit ratio in terms of thrombotic potency versus bleeding risk would be a significant improvement.

## Conclusions

**Fig. 1:**
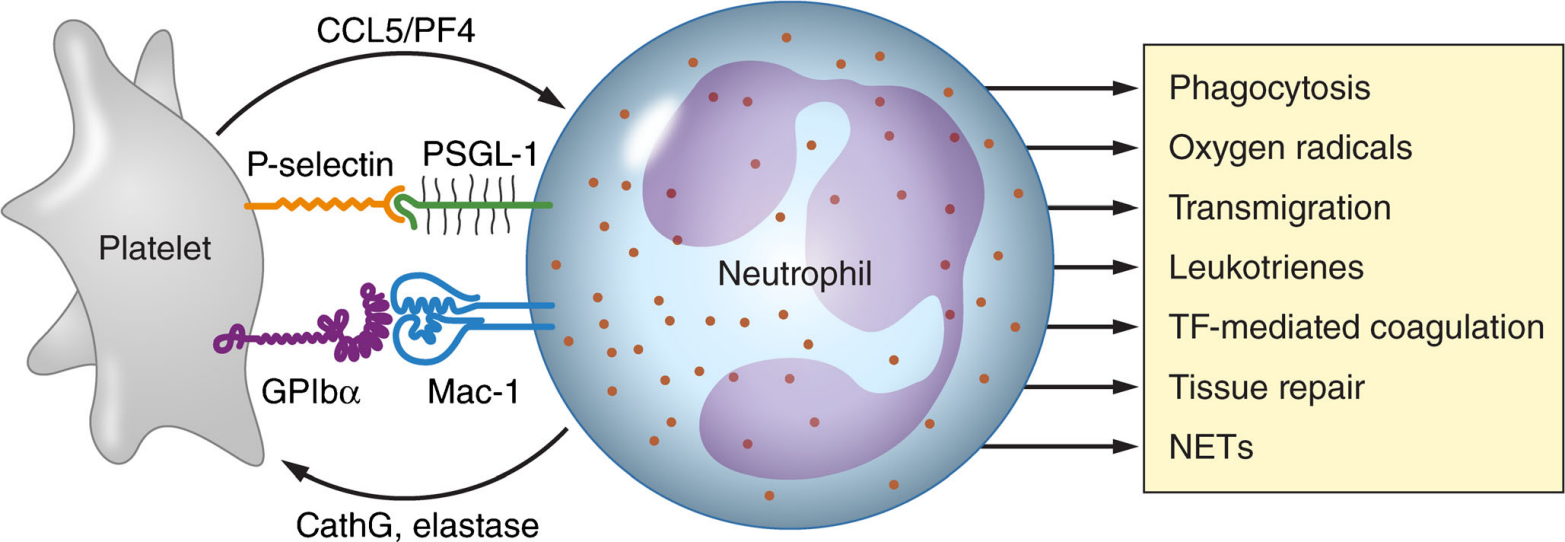
The mode of interaction of platelets and neutrophils and the consequences of this interaction. Shown are the major receptor–ligand couples involved in the platelet–neutrophil interaction (P-selectin–PSGL1 and GPIbα–Mac-1) and pathways by which platelets enhance leukocyte activation (by release of CCL5 and PF4) and by which neutrophils stimulate platelet activation (by release of elastase and cathepsin G, *CathG*). Downstream effects of the platelet–neutrophil interaction include increased leucocyte phagocytic activity, increased production of reactive oxygen species, increased transmigration of leukocytes over the endothelial cell lining, production of various bioactive leukotrienes, activation of coagulation via tissue factor (*TF*), leukocyte-mediated tissue repair and generation of neutrophil extracellular traps (*NETs*). Professional illustration by Patrick Lane, ScEYEnce Studios

Although platelets are traditionally seen as key players in thrombosis and hemostasis and neutrophils as prime inflammatory cells, recent data demonstrate a complex interplay between these cells with important immune functions for platelets and a clear role of neutrophils in thrombotic diseases. The prime mode of interaction between platelets and neutrophils and the downstream effects of this interaction are summarized in Fig. 1. The mechanisms by which the platelet–neutrophil interplay contributes to important infectious and thrombotic pathologies are only beginning to be explored, as is the role of these interactions in organ injury and repair. The discovery of NETs and the demonstration of the role of NETs in infection and thrombosis has provided clues for radical improvements in clinical strategies to combat infection, thrombosis and other diseases in which NETs are thought to play a role.
